# Clinical significance of pAkt and pErk1/2 expression in early-stage breast cancer patients treated with anthracycline-based adjuvant chemotherapy

**DOI:** 10.3892/ol.2015.2965

**Published:** 2015-02-16

**Authors:** WENJUAN LIU, LINGYUN ZHANG, JING SHI, YUNPENG LIU, LIZHONG ZHOU, KEZUO HOU, XIUJUAN QU, YUEE TENG

**Affiliations:** 1 Department of Medical Oncology, The First Hospital of China Medical University, Shenyang, Liaoning 110001, P.R. China; 2 Department of Medical Oncology, The Tumor Hospital of Anshan City, Anshan, Liaoning 114034, P.R. China

**Keywords:** breast cancer, phosphorylated Akt, phosphorylated extracellular-regulated kinase 1/2, chemotherapy-resistance, prognosis

## Abstract

The expression of phosphorylated Akt (pAkt) and phosphorylated extracellular-regulated kinase 1/2 (pErk1/2) proteins may result in breast cancer progression and drug resistance *in vitro*, however, compelling evidence regarding the clinical significance of pAkt and pErk1/2 in early-stage breast cancer is currently lacking. Thus, the aim of the present study was to determine the prognostic value of pAkt and pErk1/2 expression in early-stage breast cancer patients treated with anthracycline-based adjuvant chemotherapy. Tumor specimens were obtained from 256 patients with early-stage breast cancer who had been treated with anthracycline-based adjuvant chemotherapy, and pAkt and pErk1/2 protein expression was immunohistochemically determined. The interactions between pAkt, pErk1/2 and clinical characteristics were assessed by performing χ^2^ tests, and survival functions were estimated using the Kaplan-Meier method. It was identified that pAkt and pErk1/2 were expressed in 38.7 and 33.6% of patients, respectively, and that pAkt protein expression was correlated with pErk1/2 protein expression (P<0.001). In addition, after a median follow-up period of 52.5 months, the patients with pAkt- and pErk1/2-negative tumors experienced a significantly longer disease-free survival (DFS) time compared with pAkt- or pErk1/2-positive patients (P=0.028). pErk1/2 expression was associated with the decreased DFS time of the patients (P=0.049), and pAkt and pErk1/2 expression were associated with the decreased DFS time in human epidermal growth factor receptor (HER2)-positive patients (P=0.002). pErk1/2 expression was associated with chemotherapy resistance (P=0.016). Thus, the coexpression of pAkt and pErk1/2 was an independent factor for a poor prognosis in early-stage and HER2-positive breast cancer patients. By contrast, pErk1/2 expression alone may be a poor predictor for determining the efficacy of anthracycline-based chemotherapy.

## Introduction

The number of breast cancer patients is increasing, with >1.6×10^6^ cases diagnosed annually in China (1). Anthracycline-based chemotherapy, alone or in association with taxane administration, is considered to be a standard post-operative adjuvant treatment strategy for early-stage breast cancer (2). However, anthracycline resistance is an important problem that is often encountered during the course of adjuvant chemotherapy.

Akt, also known as protein kinase B, is a serine/threonine protein kinase that is activated by a range of stimuli via growth factor receptors, in a phosphoinositide-3-OH kinase (PI3K)-dependent manner (3). The phosphorylation of Akt has been demonstrated to promote growth factor-mediated cell growth, proliferation, migration and survival (4–6); however, it remains unclear whether the activation of Akt is associated with the poor prognosis of breast cancer patients. The results of a study conducted by Andre *et al* (7), which assessed phosphorylated Akt (pAkt) expression in 823 patients with early-stage breast cancer, indicated that the overexpression of pAkt was not correlated with patient prognosis. Furthermore, data collected from 252 breast cancer patients by Tokunaga *et al* (8) indicated that no association exists between the expression of pAkt and the disease-free survival (DFS) time of patients; however, it was identified that pAkt expression appears to predict a poor prognosis in patients treated with endocrine therapy. Additionally, Schmitz *et al* (9) collected tissue samples from 99 breast cancer patients without lymph node metastasis and determined that the expression of pAkt was associated with a shorter DFS time. The results of the aforementioned studies demonstrate discrepancies, thus, the present study proposes that the expression of pAkt may produce different effects in patients with different clinical characteristics. Furthermore, the expression of pAkt has been specifically associated with the *in vitro* resistance to doxorubicin and paclitaxel in breast cancer (10–12). It has been proposed that Akt/mammalian target of rapamycin (mTOR) pathway inhibition may sensitize breast cancer cells to doxorubicin (13,14); however, few clinical studies have investigated the association between pAkt expression and the resistance to doxorubicin in breast cancer patients.

Extracellular-regulated kinase (Erk) is a member of the mitogen-activated protein kinase (MAPK) signal-transducing family, which consists of three key cascades, termed Raf-1, Erk and p38 MAPK, with Erk being the most relevant factor in breast cancer (15). The role of Erk1 and Erk2 has been extensively studied *in vitro*; the two proteins demonstrate high structural and functional similarity, therefore, they are often referred to as Erk1/2. The intracellular activity of the MAPK pathway involves complex interactions between the PI3K/Akt cascades, which leads to proliferative and apoptotic activities being generated via competing mechanisms (16); however, it remains unclear whether augmented phosphorylated Erk1/2 (pErk1/2) activity is a prognostic factor for the outcome of breast cancer. Milde-Langosch *et al* (17) reported that high pErk1 expression was an independent indicator of a long recurrence-free survival time; however, conversely and in parallel to this study, other studies reported that increased MAPK signaling was associated with a shorter disease-free survival time (18,19). Furthermore, the expression of pErk1/2 was demonstrated to induce doxorubicin and paclitaxel resistance in breast cancer cells (15,16) By contrast, the association between pErk1/2 expression and the resistance to anthracycline in breast cancer patients remains unclear. For example, Eralp *et al* (20) reported that the expression of MAPK was associated with anthracycline resistance in triple-negative breast cancer patients, however, only 13 (11.9%) patients exhibited anthracycline-resistant disease.

Thus, the aim of the present study was to determine the clinical significance of pAkt and pErk1/2 protein expression in early-stage breast cancer patients treated with anthracycline-based adjuvant chemotherapy. Additionally, a series of hierarchical clustering analyses were conducted to determine the predictive value of pAkt and pErk1/2 for the prognosis of breast cancer patients with varying clinical characteristics.

## Materials and methods

### Patient selection and histology

A retrospective analysis was performed on 256 patients with histologically confirmed breast cancer who were treated at The First Hospital of China Medical University (Shenyang, China) or The Tumor Hospital of Anshan City (Anshan, China). The present study was approved by the Human Ethics Review Committee of the First Hospital of China Medical University, and informed consent was obtained from all patients in accordance with the Declaration of Helsinki and its later revisions. All patients underwent a mastectomy or breast conserving surgery between December 1999 and December 2008. No patients exhibited evidence of distant metastases at the time of surgery and all patients were administered with systemic adjuvant chemotherapy after surgery. The study group included 174 (68.0%) patients treated with anthracycline-based chemotherapy for 4–6 cycles every three weeks, including 5-fluorouracil (600 mg/m^2^)-epirubicin (75–80 mg/m^2^)-cyclophosphamide (600 mg/m^2^) and epirubicin (75–80 mg/m^2^)-cyclophosphamide (600 mg/m^2^) schemes, and 82 (32.0%) patients who received anthracycline associated with taxane chemotherapy for 6–8 cycles every three weeks, including epirubicin (80–100 mg/m^2^)-cyclophosphamide (600 mg/m^2^) followed by taxanes (175 mg/m^2^) and taxanes (175 mg/m^2^) followed by epirubicin(80–100 mg/m^2^)-cyclophosphamide (600 mg/m^2^) schemes. A total of 142 estrogen receptor (ER)- or progesterone receptor (PR)-positive patients received endocrine therapy with tamoxifen (20 mg, daily) for premenopausal patients and aromatase inhibitors (2.5 mg letrozole and 1 mg anastrozole, daily), for postmenopausal patients, for at least five years. Three human epidermal growth factor receptor (HER2)-positive patients received adjuvant trastuzumab (6 mg/kg) therapy every three weeks, and 12 patients received logical radiotherapy (total dose, 50 Gy), delivered to the ipsilateral chest wall, supra- and infra-clavicular lymph node regions. Routine immunohistochemical (IHC) staining was used to obtain the data regarding hormone receptor and HER2 status via analysis of the pathology reports. Additionally, medical reports were reviewed to retrieve clinical information regarding patient demographics, treatment details and outcome, and tumor samples were obtained from the patients during surgery.

### Immunohistochemical analysis and assessment

pAkt and pErk expression was assessed by performing IHC analysis on a tissue microarray containing two spots of each primary breast cancer tumor tissue. pAkt immunoreactivity, specifically the phosphorylation of serine 473, was evaluated using rabbit polyclonal antibody (Santa Cruz Biotechnology, Inc., Dallas, TX, USA) at a dilution of 1:250. For pErk1/2 detection, automatic immunostaining was performed on the DAKO Autostainer Link 48 (Dako, Glostrup, Denmark) using the monoclonal antibody phospho-p44/42 MAPK (Thr202/Tyr204), clone E10, at a dilution of 1:250 (Santa Cruz Biotechnology, Inc.). Each slide was deparaffinized in xylene and rehydrated in graded alcohol to Tris-buffered saline [50 mM Tris and 150 mM NaCl (pH 7.4)]. Antigen retrieval was performed by microwaving at 95°C for 20 min in 20 mM Tris, 10 mM citrate and 13 mM EDTA (pH 7.8). Subsequent to blocking the endogenous peroxidase activity and applying the primary antibody, the slides were incubated with biotinylated goat anti-mouse immunoglobulins (Fuzhou Maixin Biological Technology Ltd., Fujian, China) and streptavidin conjugated to horseradish peroxidase (Fuzhou Maixin Biological Technology Ltd.), with 3,3′-diaminobenzidine (DAB) chromogen solution (DAB kit; Fuzhou Maixin Biological Technology Ltd.) and a substrate buffer containing hydrogen peroxide (Fuzhou Maixin Biological Technology Ltd.) serving as the substrate system. Tissue sections were counterstained with hematoxylin, permanently mounted and independently evaluated by two individuals with no prior knowledge of the clinical data and pathological parameters. The scoring system was designed on the basis of tumor grading with respect to the ratio of stained cells to total tumor cells counted (1, 1–10%; 2, 11–33%; 3, 34–66%; and 4, 67–100%) and the intensity of cytoplasmic staining ranged from light to dark brown (0, none; 1, weak; 2, moderate; and 3, strong). By combining the grading and staining intensity scores, an expression score ranging from 0 to 7 was obtained for pAkt and pErk for each sample. Scores ranging between 0 and 3 were considered to indicate low protein expression (negative) and a score between 4 and 7 indicated a high protein expression level (positive).

### Statistical analysis

χ^2^ and Fisher’s exact tests were used to detect the association between pAkt and pErk1/2 protein expression and the clinical characteristics of the current patients. The DFS time was defined as the time between the date of surgery and the date on which the first of the following events occurred: Locoregional recurrence, distant metastasis, diagnosis of a second primary tumor or cancer-related mortality. The overall survival (OS) time was defined as the period of time between the date of surgery and the date of mortality from any cause. Furthermore, the predictive value of pAkt and pErk1/2 staining for the DFS and OS times were summarized using the Kaplan-Meier method, and compared between different arms using a stratified log-rank test. The relative risk of reducing DFS was determined using Cox proportional hazard regression with multivariate analyses. All analyses were conducted using SPSS software (version 17.0; SPSS, Inc., Chicago, IL, USA) and two-sided P<0.05 was considered to indicate a statistically significant difference.

## Results

### Patient characteristics

The median age of the cohort was 50 years (range, 26–71 years) and the majority (n=220; 85.9%) of patients exhibited invasive ductal carcinoma. The databases used in the present study were locked on August 1, 2010. After a median follow-up period of 52.5 months (range, 19–127 months), 192 patients (75.0%) were alive with no evidence of disease, 64 (25.0%) patients had relapsed and 34 (13.3%) had succumbed due to disease progression. Within 12 months of adjuvant chemotherapy, 32 (12.5%) patients developed recurrence, thus, these patients were defined as having chemotherapy-resistant disease. Detailed patient characteristics are indicated in Table I.

### pAkt and pErk1/2 expression patterns in breast cancer

**Figure 1:**
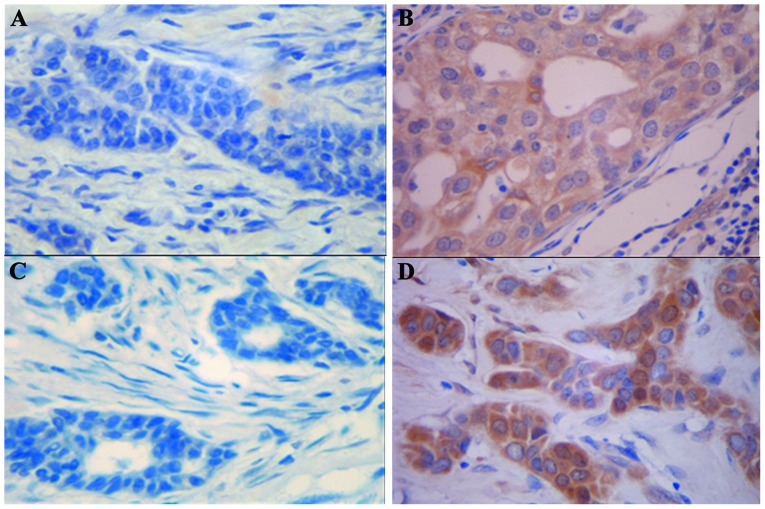
Immunohistochemical analysis, demonstrating (A) negative and (B) positive phosphorylated Akt staining; and (C) negative and (D) positive phosphorylated extracellular-regulated kinase 1/2 staining in moderately-differentiated carcinoma (magnification, ×400).

pAkt and pErk1/2 protein expression levels were evaluated in 256 breast cancer tissue samples of patients with early-stage breast cancer. Prominent cytoplasmic and partial nuclear pAkt and pErk1/2 immunoreactivity was observed in the tumor cells. In total, 99 cases (38.7%) were classified as positive for pAkt expression (Fig. 1A and B) and 86 cases (33.6%) were classified as positive for pErk1/2 expression (Fig. 1C and D); thus, a significant association was observed between the expression of pAkt and pErk1/2 (Spearman rank-correlation coefficient, r=0.284; P<0.001). The expression of pAkt was significantly correlated with the occurrence of axillary lymph node metastases (P=0.020) and with the histological grade (P=0.038). However, no statistically significant correlation was identified between pAkt expression and chemotherapy resistance (P=0.313), tumor size, patient age, or ER, PR and HER2 status. Furthermore, no correlation was identified between pErk1/2 expression and a number of patient clinical characteristics, including age, tumor size, pathology, lymph node metastases, tissue grade, and ER, PR and HER2 status. However, a statistically significant difference in positive staining for pErk1/2 was observed between chemotherapy-resistant and chemotherapy-sensitive tumors (53.1 vs. 30.8%; P=0.016). The associations between pAkt/pErk1/2 expression and the conventional clinical characteristics of the patients are shown in Table I.

### Overall prognostic value of pAkt and pErk1/2 expression in breast cancer patients

**Figure 2:**
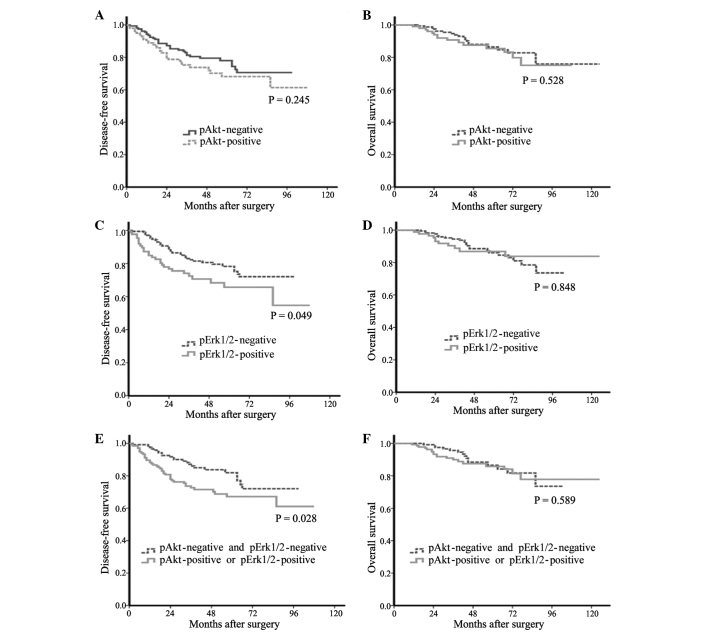
(A) Disease-free and (B) overall survival of patients with pAkt-negative versus pAkt-positive tumors. (C) Disease-free and (D) overall survival of patients with pErk1/2-negative versus pErk1/2-positive tumors. (E) Disease-free and (F) overall survival of patients with pAkt-negative and pErk1/2-negative versus pAkt-positive/pErk1/2-positive tumors. pAkt, phosphorylated Akt; pEkr1/2, phosphorylated extracellular-regulated kinase 1/2.

After a median follow-up period of 52.5 months, the data indicated that the expression of pAkt was not significantly associated with the DFS or OS times of the patients (P=0.245 and P=0.528, respectively; Fig. 2A and B). The expression of pErk1/2 was significantly associated with a shorter DFS time (P=0.049, Fig. 2C), however, no significant difference was identified in the OS time (P=0.848; Fig. 2D). By contrast, pAkt- or pErk1/2-positive tumors were significantly associated with a decreased DFS time compared with pAkt- and pErk1/2-negative tumors (P=0.028; Fig. 2E), however, no significant difference was observed in the OS time (P=0.171; Fig. 2F). Subsequently, Cox regression with multivariate analysis was performed to determine the independent prognostic value of different variables in association with DFS time, indicating that the following factors significantly contribute to a decrease in DFS: Positive lymph node metastasis (P=0.001), ER/PR-negative tumors (P=0.030) and the coexpression of pAkt and pErk1/2 (P=0.032) (Table II).

### Predictive value of pAkt and pErk1/2 expression for the DFS time of patients in various subgroups

Recent studies have indicated that breast cancer patients with specific tumor subtypes may be more resistant to therapy and therefore exhibit decreased DFS times (21–24). Thus, the present study examined the effect of pAkt and pErk1/2 expression on the following tumor subgroups: HER2-positive; HER2-negative; ER- or PR-positive; ER- and PR-negative; lymph node-positive; and lymph node-negative subgroups. In the HER2-positive subgroup, the DFS time of the pAkt-negative patients was significantly longer than that of the pAkt-positive patients (P=0.002); additionally, a significant association was identified between pErk1/2 expression and decreased DFS time (P=0.002). pAkt and pErk1/2 expression was not significantly associated with the DFS time of the patients in the HER2-negative (P=0.623 and P=0.563, respectively), and ER- and PR-positive (P=0.726 and P=0.223, respectively) subgroups. In the ER- and PR-negative subgroup, no significant association was observed between the expression of pErk1/2 and the DFS time of the patients (P=0.118); however, the DFS time of the pAkt-negative patients was significantly longer than that of the pAkt-positive patients (P=0.034). Furthermore, pAkt and pErk1/2 expression was not significantly associated with the DFS time of the patients in the subgroup with lymph node metastases (P=0.778 and P=0.345, respectively) or the lymph node-negative subgroup (P=0.245 and P=0.123, respectively) (Table III).

### Predictive values of pAkt and pErk1/2 expression to determine the efficacy of anthracycline-based chemotherapy

In the patients treated with anthracycline-based chemotherapy, pAkt and pErk1/2 expression was not associated with DFS of patients (P=0.238 and P=0.382, respectively). In the patients treated with anthracycline associated with taxane chemotherapy, no correlation was identified between the expression of pAkt and the DFS time of the patients (P=0.743), however, the DFS time of the pErk1/2-negative patients was significantly longer than that of the pErk1/2-positive patients (P=0.030) (Table III).

## Discussion

The present study examined the expression of pAkt and pErk1/2 in 256 patients with early-stage breast cancer by performing IHC analysis of the tumor samples. pAkt and pErk1/2 protein expression was identified in 38.7 and 33.6% of the tissue samples, respectively, and the cytoplasmic and nuclear immunoreactivity of pAkt and pErk1/2 was observed in the breast cancer cells. The mechanisms that regulate the induction of Akt appear to be involved in the activation of Erk1/2 (25), however, no large-scale clinical study has been conducted analyzing the association between pAkt and pErk1/2 expression. The results of the present study indicated that a significant positive correlation exists between the expression of pAkt and pErk1/2 proteins in breast cancer patients. Furthermore, pAkt expression was associated with positive nodal status and histological grade, however, no significant correlation was identified between pAkt and tumor size, indicating that pAkt may induce a more malignant phenotype via its role in antiapoptosis and proliferation. By contrast, no correlation was observed between pErk1/2 expression and conventional clinical patient characteristics.

Although the activation of Akt appears to be a potentially major event in the survival of breast cancer cells, the present study did not demonstrate a significant association between pAkt protein expression and the prognosis of patients with early-stage breast cancer; this is consistent with the results found by Andre *et al* (7). Previous clinical studies regarding the prognostic value of pErk1/2 expression in patients with early-stage breast cancer have produced discrepancies. For example, the expression of pErk1/2 was identified to positively correlate with an increased risk of relapse (18), which is in contrast to the results determined by Milde-Langosch *et al* (18), where high expression of pErk1/2 was significantly associated with a long recurrence-free survival time. The present study demonstrated that pErk1/2 expression was associated with a poor prognosis in 256 patients with early-stage breast cancer. Additionally, to the best of our knowledge, the present study is the first to report that the coexpression of pAkt and pErk1/2 are predictive of a shorter DFS time in early-stage breast cancer patients.

The overexpression of HER2 occurs in ~30% of cases of human breast cancer and is typically associated with a poor prognosis (26–28). pAkt and pErk1/2 are important downstream substrates of HER2 (25–35). Morse *et al* (28) demonstrated that the inhibition of HER2 signaling may decrease pErk and pAkt expression levels, and reduce breast tumor cell proliferation. Furthermore, Tokunaga *et al* (8) reported that HER2-/pAkt-positive tumors appear to exhibit a trend for a poorer prognosis, and Park *et al* (30) indicated that increased cytoplasmic and nuclear pAkt concentrations may significantly correlate with HER-2 overexpression. Similarly, the present study identified that pAkt expression was significantly associated with decreased DFS time in the HER2-positive patients. As the phosphorylation of Akt can induce the resistance of breast cancer to trastuzumab treatment (31,32), the results of the current study indicate that the treatment of HER2-positive tumors with pAkt inhibitors may aid in overcoming trastuzumab resistance. It has previously been reported that a higher level of pErk1/2 expression predicts a shorter DFS time in lymph node-positive/HER2-positive patients (33). Similarly, the present study indicated that the expression of pErk1/2 was associated with a shorter DFS time in HER2-positive patients with early-stage breast cancer. Thus, concurrent pAkt and pErk expression may be predictive of a poor prognosis in HER2-positive patients, and a dual target inhibitor of pAkt and pErk1/2 may be a potential effective therapeutic approach for the treatment of HER2-positive breast cancer, overcoming the resistance of breast cancer patients to trastuzumab therapy.

Previous studies have identified a specific association between the expression of pAkt and pErk1/2, and the proliferation and chemoresistance of breast cancer cells *in vitro*. Additionally, a number of studies have reported that anthracycline induces secondary Akt activation, which may contribute to the resistance of breast cancer cells to anthracycline *in vitro* (10–12). Inhibitors of the PI3K/Akt/mTOR pathways, such as rapamycin analogs, may be used as a therapeutic approach to modulate anthracycline-induced Akt activation. However, similar to the results found by Andre *et al* (7), pAkt expression did not appear to be predictive for anthracycline efficacy in the present study and was not associated with anthracycline resistance. Furthermore, the Raf/MAPK kinase/Erk pathway has been demonstrated to induce resistance to doxorubicin and paclitaxel via ectopic activation of Raf in breast cancer cells (15,16). Similarly, the present study confirmed that a significant correlation exists between pErk1/2 and chemotherapy-resistance; this result is in accordance with a study conducted by Eralp *et al* (20), which determined that pErk1/2 expression was associated with anthracycline resistance in triple-negative breast cancer patients. Additionally, the present study indicated that pErk1/2 expression was a poor predictor for the efficacy of adjuvant anthracycline-based chemotherapy, particularly for patients treated with anthracycline associated with taxane chemotherapy. Thus, the inhibition of pErk1/2 may be an effective therapeutic approach to modulate anthracycline resistance in breast cancer.

## Figures and Tables

**Table I:** Correlation between pAkt and pErk1/2 expression, and the clinicopathological parameters in breast cancer patients.

		pAkt expression			pErk1/2 expression		
			
Parameter	Patients, n	−, n (%)	+, n (%)	P-value^a^	−, n (%)	+, n (%)	P-value^a^
Age, years				0.054			1.000
≤50	132	73 (55.3)	59 (44.7)		88 (66.7)	44 (33.3)	
>50	124	84 (67.7)	40 (32.3)		82 (66.1)	42 (33.9)	
Tumor size, cm				0.886			0.460
≤2	70	42 (60.0)	28 (40.0)		44 (62.9)	26 (37.1)	
>2	184	113 (61.4)	71 (38.6)		125 (67.9)	59 (32.1)	
Lymph node involvement				0.020^b^			0.286
0	111	77 (69.4)	34 (30.6)		78 (70.3)	33 (29.7)	
≥1	143	78 (54.5)	65 (45.5)		91 (63.6)	52 (36.4)	
Histological grade				0.038^b^			0.624
I	18	14 (77.8)	4 (22.2)		11 (61.1)	7 (38.9)	
II	146	80 (54.8)	66 (45.2)		96 (65.8)	50 (34.2)	
III	63	44 (69.8)	19 (30.2)		45 (71.4)	18 (28.6)	
ER				0.365			0.592
0	146	93 (63.7)	53 (36.3)		95 (65.1)	51 (34.9)	
1–3	109	63 (57.8)	46 (42.2)		75 (68.8)	34 (31.2)	
PR				0.519			0.894
0	139	88 (63.3)	51 (36.7)		92 (66.2)	47 (33.8)	
1–3	116	68 (58.6)	48 (41.4)		78 (67.2)	38 (32.8)	
HER2				0.414			0.676
0–1	156	93 (59.6)	63 (40.4)		105 (67.3)	51 (32.7)	
2–4	90	59 (65.6)	31 (34.4)		58 (64.4)	32 (35.6)	
Chemotherapy resistance				0.564			0.016^a^
Yes	32	18 (56.2)	14 (43.8)		15 (46.9)	17 (53.1)	
No	224	139 (62.1)	85 (37.9)		155 (69.2)	69 (30.8)	

a Determined by χ^2^ test;

b P<0.05.

pAkt, phosphorylated Akt; pEkr1/2, phosphorylated extracellular-regulated kinase 1/2; ER, estrogen receptor; PR, progesterone receptor; HER2, human epidermal growth factor receptor.

**Table II:** Results of multivariate Cox regression analysis to determine the independent prognostic value of different variables in association with DFS time.

Covariate	Relative risk	95% CI	P-value
Age, years (>50 vs. <50)	1.013	0.603–1.703	0.961
Tumor size, cm (>2 vs. ≤2)	1.118	0.587–2.130	0.733
Lymph node involvement (positive vs. negative)	3.079	1.612–5.880	0.001^a^
Hormone status (positive vs. negative)	0.563	0.335–0.947	0.030^a^
HER2 status (positive vs. negative)	0.927	0.539–1.598	0.785
Coexpression of pAkt and pErk1/2 (positive vs. negative)	1.817	1.015–3.139	0.032^a^

a P<0.05.

DFS, disease-free survival; CI, confidence interval; HER2, human epidermal growth factor receptor; pAkt, phosphorylated Akt; pErk1/2, phosphorylated extracellular-regulated kinase 1/2.

**Table III:** Univariate analysis of pAkt and pErk1/2 for predictive DFS values in different subgroups.

	P-value^a^	
	
Patient subgroup	pAkt expression	pErk1/2 expression
HER2-positive (n=90)	0.002^b^	0.002^b^
HER2-negative (n=156)	0.623	0.563
ER- or PR-positive (n=141)	0.726	0.223
ER- and PR-negative (n=114)	0.034^b^	0.118
Lymph node-positive (n=143)	0.788	0.345
Lymph node-negative (n=111)	0.245	0.123
Anthracycline-based chemotherapy (n=174)	0.238	0.382
Anthracycline and taxane chemotherapy (n=82)	0.743	0.030^b^

a Determined by Kaplan-Meier and log-rank tests;

b P<0.05.

pAkt, phosphorylated Akt; pEkr1/2, phosphorylated extracellular-regulated kinase 1/2; DFS, disease-free survival; HER2, human epidermal growth factor receptor; ER, estrogen receptor; PR, progesterone receptor.
